# Cholesteryl Ester Transfer Protein Inhibition for Preventing Cardiovascular Events: *JACC* Review Topic of the Week

**DOI:** 10.1016/j.jacc.2018.10.072

**Published:** 2019-02-05

**Authors:** Jane Armitage, Michael V. Holmes, David Preiss

**Affiliations:** Medical Research Council Population Health Research Unit, Clinical Trial Service Unit and Epidemiological Studies Unit, Nuffield Department of Population Health, University of Oxford, Oxford, United Kingdom

**Keywords:** cardiovascular disease, CETP, CETP inhibitor, HDL cholesterol, LDL cholesterol, Mendelian randomization, randomized trial, BP, blood pressure, CE, cholesteryl ester, CETP, cholesteryl ester transfer protein, GWAS, genome wide association study/studies, HDL, high-density lipoprotein, HR, hazard ratio, LDL, low-density lipoprotein, PTV, protein-truncating variant, TG, triglycerides

## Abstract

Cholesteryl ester transfer protein (CETP) facilitates exchange of triglycerides and cholesteryl ester between high-density lipoprotein (HDL) and apolipoprotein B100–containing lipoproteins. Evidence from genetic studies that variants in the *CETP* gene were associated with higher blood HDL cholesterol, lower low-density lipoprotein cholesterol, and lower risk of coronary heart disease suggested that pharmacological inhibition of CETP may be beneficial. To date, 4 CETP inhibitors have entered phase 3 cardiovascular outcome trials. Torcetrapib was withdrawn due to unanticipated off-target effects that increased risk of death, and major trials of dalcetrapib and evacetrapib were terminated early for futility. In the 30,000-patient REVEAL (Randomized Evaluation of the Effects of Anacetrapib through Lipid Modification) trial, anacetrapib doubled HDL cholesterol, reduced non-HDL cholesterol by 17 mg/dl (0.44 mmol/l), and reduced major vascular events by 9% over 4 years, but anaceptrapib was found to accumulate in adipose tissue, and regulatory approval is not being sought. Therefore, despite considerable initial promise, CETP inhibition provides insufficient cardiovascular benefit for routine use.

Since the 1990 discovery in Japan of individuals homozygous for mutations in *CETP* who displayed no measurable cholesteryl ester transfer protein (CETP) along with substantially elevated high-density lipoprotein cholesterol (HDL-C) and modestly reduced low-density lipoprotein cholesterol (LDL-C), (1) there has been substantial interest in CETP as a pharmacological target to reduce the incidence of cardiovascular disease (Central Illustration).

**Central Illustration:**
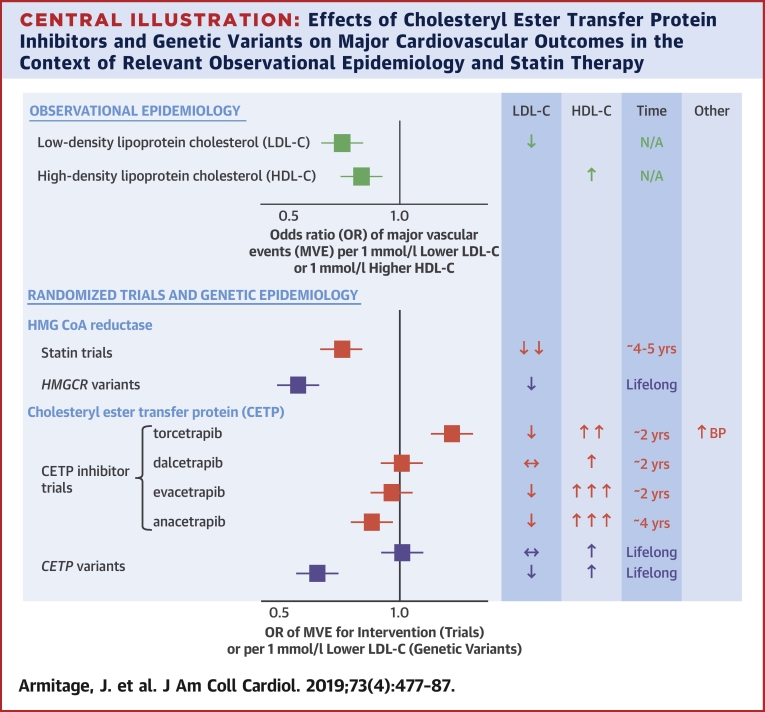
Effects of Cholesteryl Ester Transfer Protein Inhibitors and Genetic Variants on Major Cardiovascular Outcomes in the Context of Relevant Observational Epidemiology and Statin Therapy ↑ = increase; ↓ = decrease; ↔ = unchanged; BP = blood pressure; CETP = cholesteryl ester transfer protein; HDL-C = high-density lipoprotein cholesterol; HMG CoA = 3-hydroxy-3-methyl-glutaryl-coenzyme A; HMGCR = HMG CoA reductase; LDL-C = low-density lipoprotein cholesterol; MVE = major vascular events; N/A = not available; OR = odds ratio.

## The Biology of CETP

**Figure 1:**
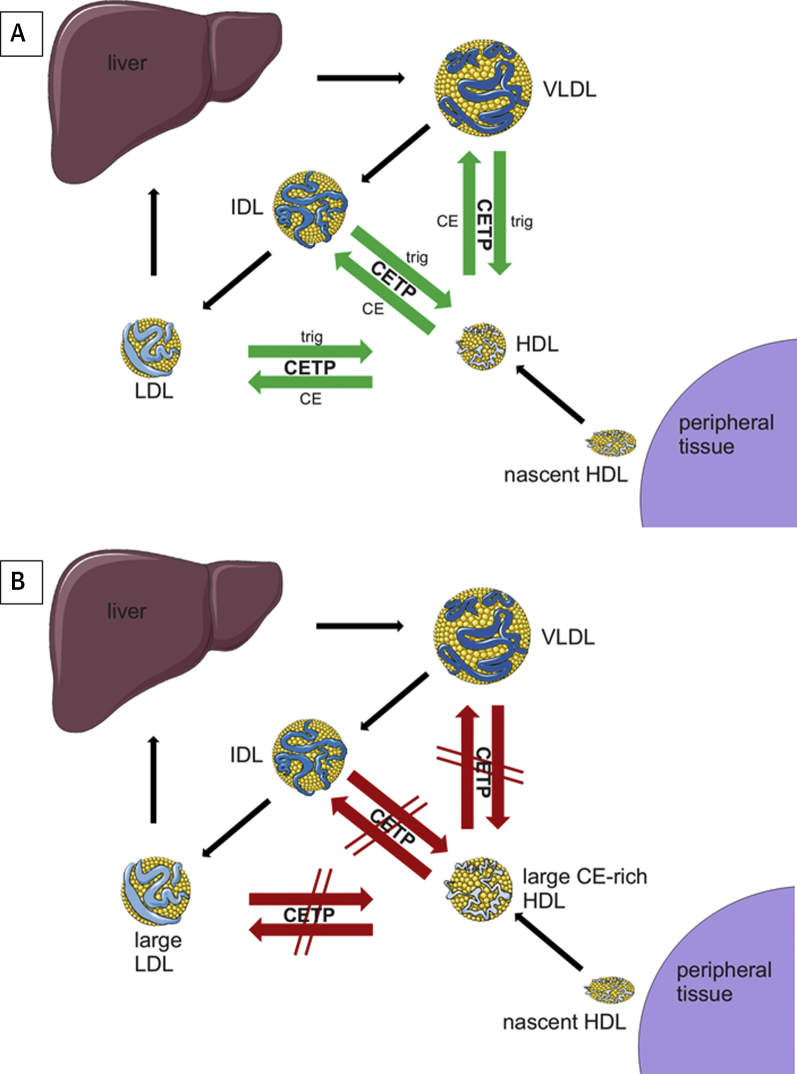
The role of CETP in Lipid Metabolism and the Effect of CETP Inhibition on Circulating Lipoproteins **(A)** CETP in lipid metabolism and **(B)** the effect of CETP inhibition on circulating lipoprotein. CE = cholesteryl ester; CETP = cholesteryl ester transfer protein, HDL = high-density lipoprotein; IDL = intermediate-density lipoprotein; LDL = low-density lipoprotein; trig = triglycerides; VLDL = very low-density lipoprotein.

CETP is found in the circulation mainly bound to high-density lipoprotein (HDL). CETP allows equimolar transfer of neutral lipids (cholesterol esters [CE] and triglycerides [TG]) between plasma HDL and apolipoprotein B100–containing lipoprotein particles (Figure 1). The net effect of CETP is to transport CE from HDL to both very low-density lipoprotein (VLDL) and low-density lipoprotein (LDL), with TG moving in the opposite direction. The precise explanation for how CETP transfers neutral (i.e., no net charge) lipid between lipoproteins is not fully resolved. The commonly accepted hypothesis is that molecular forces lead to twisting and opening of a tunnel within the CETP molecule through which CE and TG can transfer 2, 3. According to this “tunnel mechanism” theory, bound CEs in the core of the CETP molecule change their shapes between bent and linear conformations, and these changes together lead to the spontaneous formation of a continuous tunnel across the entire length of the CETP molecule. However, other studies have reached different conclusions, namely that either terminal (N or C) may bind to HDL and that a ternary structure and the presence of a tunnel is not necessarily required to explain CETP’s function (4).

## Evidence From Genetic Studies That CETP Is Causally Related to Cardiovascular Disease

### Genome-wide association studies of CETP, blood lipids, and coronary heart disease

The *CETP* gene is located on chromosome 16 and consists of ∼22 kilo base pairs with 16 exons. Genome-wide association studies (GWAS) 5, 6 of blood lipids measured in >100,000 individuals have identified rs3764261 in *CETP* to be associated with higher HDL and total cholesterol, and lower LDL cholesterol and TG, all at p ≤ 2 × 10^−25^. Furthermore, GWAS of 42,335 individuals with coronary heart disease (CHD) and 78,240 control subjects identified a variant in *CETP* (rs1800775) associated with higher risk of CHD (p = 9.8 × 10^−9^) (7), and also with lower HDL-C, higher LDL-C, and higher TG.

### Large prospective cohorts (>10,000 individuals), CETP variants, and risk of CHD

In 2000, Agerholm-Larsen et al. (8) investigated 2 common variants in *CETP* (A373P and R451Q) in ∼10,000 Danish individuals and found that while these variants had strong associations with HDL-C, they were not associated with apolipoprotein-B concentrations or risk of CHD (Table 1). In 2009, Ridker et al. (9) took a hypothesis-free approach to identify SNPs associated with HDL-C in 18,245 women from the Women’s Genome Health study, identifying 20 SNPs in/around *CETP* associated with HDL-C at GWAS significance that associated with risk of incident myocardial infarction. Johannsen et al. (10) subsequently quantified 2 common variants in *CETP* in 10,261 individuals from the Copenhagen City Heart Study and found that combining the variants led to higher associations with HDL-C, lower TG and non−HDL-C, and lower risks of ischemic vascular events, including myocardial infarction and ischemic stroke.

Thompson et al. (11) meta-analyzed data from 102 studies published between 1970 and 2008 with ≤147,599 individuals of Caucasian and East Asian descent and up to 27,196 CHD cases, and reported the association of 3 common variants (TaqIB rs708272, I405V rs5882, and −629C>A rs1800775) within the *CETP* locus with CETP mass and activity, blood lipid concentrations, and risk of CHD. The 3 variants, when orientated to a higher HDL-C, had weak associations with a lower risk of CHD, with odds ratios (ORs) of 0.95 (95% confidence interval [CI]: 0.92 to 0.99) for rs708272; 0.94 (95% CI: 0.89 to 1.00) for rs5882, and 0.95 (95% CI: 0.91 to 1.00) for rs1800775.

Nomura et al. (12) sequenced exons of *CETP* in 58,469 individuals from 12 case-control studies (18,817 CHD cases and 39,652 control subjects) to identify protein-truncating variants (PTVs). Individuals carrying 1 *CETP* PTV, of whom there were 60 in the study, had 22.6-mg/dl (0.59-mmol/l) higher HDL-C, 12.2-mg/dl (0.32-mmol/l) lower LDL-C, and 6.3% lower TG. Pooling the associations across the individual studies, including data from non-European studies, the summary association of *CETP* PTVs with risk of CHD was an OR of 0.70 (95% CI: 0.54 to 0.90).

In a study reported just prior to the findings of REVEAL (Randomized Evaluation of the Effects of Anacetrapib through Lipid Modification) being published (13), investigators sought to anticipate the effects of CETP inhibitor therapy on the background of statin treatment. Using a factorial Mendelian randomization design, Ference et al. (13) used data from 102,837 individuals with 13,821 major vascular events. Weighted genetic instruments were constructed from 8 SNPs in and around *CETP* that associated with HDL-C levels at GWAS significance, with the genetic instrument dichotomized at the median to approximate random allocation to a CETP inhibitor in a randomized trial. A similar process was performed for 6 SNPs in/around *HMGCR* that associated with LDL-C. Individuals with higher *CETP* gene scores (proxying therapeutic inhibition of CETP) had a lower risk of major vascular events (OR: 0.964; 95% CI: 0.955 to 0.983). There was clear evidence of a dose-response relationship, with the *CETP* alleles conferring higher HDL-C concentrations and lower LDL-C, plus apolipoprotein B100 having a monotonic association with lower risk of major vascular events. In a factorial Mendelian randomization analysis, individuals with low *HMGCR* and high *CETP* scores (proxying treatment with a CETP inhibitor in the absence of statin therapy) had a lower risk of major vascular events (OR: 0.946; 95% CI: 0.921 to 0.972). However, the same comparison of *CETP* among those with high *HMGCR* score (proxying CETP inhibitor treatment in the presence of statin treatment) had a slightly weaker association with major vascular events (OR: 0.985; 95% CI: 0.959 to 1.012). The authors noted in this latter analysis that there was a discrepancy in the reductions of LDL-C and apolipoprotein B100, with a smaller reduction in apolipoprotein B100 than LDL-C. The authors anticipated that treatment with a CETP inhibitor on the background of statin therapy would lead to a reduction in cardiovascular risk in proportion to the reduction in apolipoprotein B100 (which might be smaller than the reduction in LDL-C).

Millwood et al. (14) selected 5 *CETP* variants (including rs2303790, a loss of function variant) genotyped in >150,000 participants of the China Kadoorie Biobank with 24,373 incident major vascular events. A gene score containing the 5 variants had strong positive associations with HDL-C, a weaker negative effect on TG, and, interestingly, a weak effect on LDL-C in the opposite direction to the association identified in Europeans. Nuclear magnetic resonance metabolomics showed higher esterified cholesterol in HDL particles and higher TG within VLDL particles, confirming reduced CETP activity. The *CETP* gene score showed no relationship with risk of major vascular events: OR: 0.97 (95% CI: 0.91 to 1.04).

### Summation of genetic data

**Figure 2:**
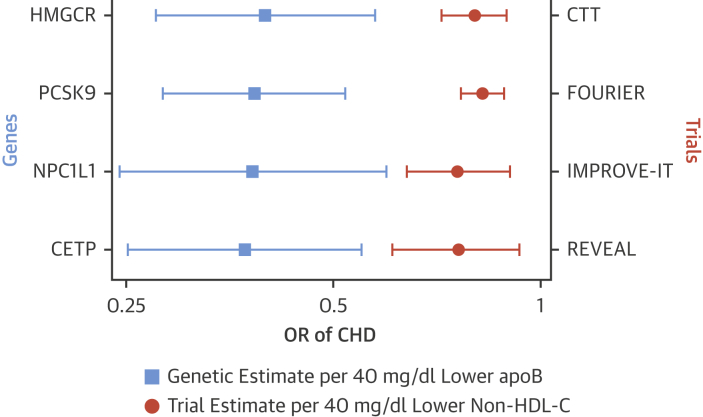
Associations of Clinical Trials of Lipid-Modifying Therapies, the Corresponding Genetic Proxies, and Risk of Coronary Heart Disease Clinical trials results are scaled to a 40-mg/dl lower non–HDL cholesterol and genetic associations are scaled to a 40-mg/dl lower apolipoprotein B100. Clinical trial data are taken from the original trials, with the exception of the CTT estimate, which is derived from Supplementary Figure 5 of the REVEAL trial. Genetic data are obtained from Ference et al. (13). Endpoints for the clinical trials are: 1) REVEAL: myocardial infarction or coronary death; 2) FOURIER: myocardial infarction; 3) CTT: myocardial infarction or coronary death; and 4) IMPROVE-IT: myocardial infarction. Endpoints for the genetic estimates are myocardial infarction, coronary death, coronary revascularization, or stroke. apo B = apolipoprotein B100; CTT = Cholesterol Treatment Trialists Collaboration; HMGCR = HMG CoA reductase; NPC1L1 = Niemann-Pick C1-Like 1; OR = odds ratio; PCSK9 = Proprotein Convertase Subtilisin/Kexin type 9; other abbreviations as in Figure 1.

SNPs in the *CETP* gene have been identified that influence the major blood lipid traits and associate with risk of CHD, all at GWAS significance. This provides some evidence that, provided these SNPs are valid proxies, CETP inhibitor therapy may lead to a reduction in risk of CHD. Two notable findings are that the magnitude of the *CETP* to CHD signal from genetic studies appear to be consistent with the non-HDL-C (or apolipoprotein B100) association of *CETP* SNPs (Figure 2) and that *CETP* SNPs with no effect on apolipoprotein B100 have no effect on cardiovascular risk.

## Pharmacological Inhibition of CETP

### Animal models of CETP inhibition

Various studies have been conducted in animal models, most notably rabbits, which have comparable CETP activity to humans. Approaches have included antisense oligonucleotides and vaccination against CETP plus treatment with some CETP inhibitors that were subsequently tested in man. These approaches yielded impressive increases in HDL-C, moderate reductions in LDL-C, and improvements in surrogates of cardiovascular disease such as aortic arch atherosclerosis. Although moderately encouraging, the possibility of publication bias and the relevance of rabbits to human physiology were highlighted as reasons to be cautious about extrapolating these results to humans (15).

### Randomized controlled trials of CETP inhibitors in man

The first published reports of a small molecule (PD 140195) CETP inhibitor occurred in 1994 (16), and a variety of inhibitors were isolated and tested during the late 1990s. CETP inhibition increases HDL-C and, for more potent inhibitors, decreases LDL-C and apolipoprotein B100 (Figure 1). Additional effects include increased cholesterol efflux, significant increases in the TG/cholesterol ratio of apolipoprotein B100-carrying particles, and reduced production and levels of lipoprotein(a).

Four CETP inhibitors, namely torcetrapib, dalcetrapib, evacetrapib, and anacetrapib, eventually entered phase 3 cardiovascular outcome trials (Table 2).

**Table 1:** Prospective Cohort Studies of CETP Variants and Risk of Vascular Disease

First Author (Ref. #)	Study Name	Outcome	Cases/Total Sample Size	CETP Variants	Main Findings
Agerholm-Larsen et al. (8)	Copenhagen City Heart Study	IHD	698/9,166	A373P and R451Q mutations	CETP variants associated with higher HDL-C but not with IHD
Ridker et al. (9)	Women’s Genome Health study	MI	198/18,245	Several SNPs in *CETP* locus	CETP variants associated with higher HDL-C and lower risk of MI
Johannsen et al. (10)	Copenhagen City Heart Study	Ischemic vascular disease	2,743/10,261	2 common variants (rs1800775 and rs708272)	CETP variants associated with higher HDL-C and lower risk of ischemic vascular disease
Millwood et al. (14)	China Kadoorie Biobank	MVE	24,373/151,217	5 variants including loss of function rs2303790	CETP variants associated with higher HDL-C but not lower LDL-C. No association with MVE

CETP = cholesteryl ester transfer protein; HDL-C = high-density lipoprotein cholesterol; IHD = ischemic heart disease; LDL-C = low-density lipoprotein cholesterol; MI = myocardial infarction; MVE = major vascular events; SNP = single nucleotide polymorphisms.

#### Torcetrapib

Torcetrapib binds reversibly to CETP and is a noncompetitive inhibitor, which induces the formation of a stable complex between CETP and HDL. An early multidose study in healthy volunteers showed marked dose-dependent increases in HDL-C up to 90%, with up to 42% reductions (estimated by the Friedewald equation) in LDL-C, and a 26% reduction in apolipoprotein B (17). At the maximum tested dose there was over 80% inhibition of CETP activity.

RADIANCE (Rating Atherosclerotic Disease Change by Imaging with a New CETP Inhibitor) 1 randomized 900 patients with heterozygous familial hypercholesterolemia with elevated baseline lipid levels (18) to 60 mg torcetrapib or placebo daily along with background atorvastatin therapy, and carotid intima thickness was assessed over 24 months by B-mode ultrasound. Lipid effects showed an increase in HDL-C of 52% and LDL-C reduction (Friedewald formula) of 21%, but systolic blood pressure (BP) was noted to increase by 2.8 mm Hg (p < 0.001) on torcetrapib. There was no significant difference in carotid intima-media thickness across 12 arterial sites but an increase in intima-media thickness of the common carotid artery (a secondary endpoint), suggesting possibly worsening atherosclerosis with torcetrapib. RADIANCE 2, conducted in 752 patients with mixed dyslipidemia randomized to atorvastatin plus 60 mg torcetrapib daily versus atorvastatin plus placebo, also showed no significant difference in measures of carotid intima-media thickness, but again a mean increase in systolic BP of 5.4 mm Hg on torcetrapib versus placebo (19). Both trials were conducted while the larger clinical outcome study, ILLUMINATE (Investigation of Lipid Level Management to Understand Its Impact in Atherosclerotic Events), was ongoing, but published after it had closed.

The ILLUMINATE study randomized 15,067 patients at high cardiovascular risk to torcetrapib 60 mg daily plus atorvastatin or atorvastatin plus placebo from 2004 to 2005 (20). Effects on lipids included a 72% increase in HDL-C and 25% decrease in LDL-C (Friedewald formula). The primary endpoint was a composite of death from coronary heart disease, nonfatal myocardial infarction stroke, or hospitalization for angina. The trial was stopped prematurely because more deaths were observed among those receiving torcetrapib than placebo (93 vs. 59) with excesses of both cardiovascular and noncardiovascular mortality. The primary outcome was also significantly more common among those allocated torcetrapib (6.2% vs. 5.0%; hazard ratio [HR]: 1.25; 95% CI: 1.09 to 1.44). There was again an increase in systolic BP of about 5 mm Hg. Post hoc analyses attribute the increase in BP to off-target effects of increased aldosterone leading to electrolyte changes and related metabolic effects (20). The results of ILLUMINATE led to the termination of torcetrapib’s development. Torcetrapib improved glycemic control in diabetes and produced consistent effects on glycated hemoglobin in people without diabetes (21).

#### Dalcetrapib

A CETP inhibitor molecule known as JTT-705 and shown to decrease atherosclerosis in rabbits was found to inhibit CETP activity by 30% to 40%. In a phase 2 study in 198 healthy subjects, JTT-705 reduced CETP activity by 37%, increased HDL-C by 34%, and reduced LDL-C by 7% (22). In 2004, Roche acquired the rights to develop JTT-705 and it was named dalcetrapib. Dalcetrapib is a noncompetitive inhibitor that binds irreversibly to CETP via formation of a covalent disulﬁde bond, a feature not found with the other 3 inhibitors.

Dal-VESSEL randomized 476 subjects to dalcetrapib 600 mg daily versus placebo added to usual therapy including statins for 36 weeks (23). Coprimary endpoints were brachial artery endothelial function assessed by flow-meditated dilatation and 24-h ambulatory BP. There was no change in flow-meditated dilatation either at 12 or 36 weeks and no effect on BP at any time point. Lipid effects revealed increases in HDL-C of 25% to 31%, but no reduction in LDL-C. The Dal-PLAQUE study assessed dalcetrapib’s impact on magnetic resonance imaging arterial indexes and PET/CT inflammatory endpoints over 24 months (24). A total of 130 patients with or at risk of vascular disease were randomized to dalcetrapib 600 mg daily or placebo. Carotid imaging results showed no effect on plaque burden, and 1 of several coprimary endpoints was suggestive of benefit with total vessel area increasing less with dalcetrapib than placebo. From 2008 to 2010, the Dal-OUTCOMES trial randomized 15,871 patients within 12 weeks of experiencing an acute coronary syndrome to dalcetrapib or placebo (25). The primary endpoint was a composite of death from coronary heart disease, nonfatal myocardial infarction, ischemic stroke, unstable angina, or cardiac arrest. After median follow-up of 31 months, the trial was stopped for futility. Compared with placebo, HDL-C increased by about 25% on dalcetrapib, and there was no effect on LDL-C or apolipoprotein B. The primary endpoint occurred in 8.3% on dalcetrapib versus 8.0% on placebo (HR: 1.04; 95% CI: 0.93 to 1.16). There was a small increase in systolic BP of 0.6 mm Hg.

A speculative, subgroup post hoc pharmacogenomics analysis showed that the presence of SNPs in the *ADCY9* gene on chromosome 16 were associated with favorable effects on cardiovascular events and carotid intima-media thickness progression with dalcetrapib (26). More than 40% of participants in Dal-OUTCOMES carried an apparently protective allele, while 20% were homozygous for protective alleles. Although it is unclear how ADCY9 might influence dalcetrapib’s effect, this finding has formed the rationale for the ongoing dal-GenE (Effect of Dalcetrapib vs Placebo on CV Risk in a Genetically Defined Population With a Recent ACS) trial in which dalcetrapib is being tested in a genetically defined population (NCT02525939).

#### Evacetrapib

Evacetrapib is a potent, selective, and reversible CETP inhibitor. Early studies in man showed dose-dependent increases in HDL-C of up to 130% and LDL-C reductions (measured by enzymatic methods) ≤40% with monotherapy (27), and up to a 90% increase in HDL-C and 14% additional reduction in LDL-C in combination with statins. With up to 600 mg daily of evacetrapib, no effect was seen on ambulatory BP or on biomarkers of renin angiotensin activation (28).

The ACCELERATE (Assessment of Clinical Effects of Cholesteryl Ester Transfer Protein Inhibition with Evacetrapib in Patients at a High Risk for Vascular Outcomes) trial randomized 12,092 patients with vascular disease during 2012 to 2013 (29). Participants had a history of acute coronary syndrome, cerebrovascular disease, peripheral artery disease, or diabetes with coronary artery disease. Patients received evacetrapib 130 mg daily or placebo on top of usual care with 96% on statins. The primary outcome was a composite of death from cardiovascular disease, myocardial infarction, stroke, coronary revascularization, or hospitalization for unstable angina. After 3 months, HDL-C had risen by 132%, and LDL-C (measured by beta quantification [personal communication from trialists, Professor Stephen Nicholls, December 21, 2017]) was reduced by 37% (29 mg/dl [0.76 mmol/l]) versus placebo, a larger reduction than expected given the modest 19% reduction in apolipoprotein B100. Based on a more likely reduction in LDL-C of approximately 19% (15 mg/dl [0.40mmol/l]) and bearing in mind that cardiovascular benefit in the first year of statin treatment is perhaps one-half that observed in later years, a 9% reduction in cardiovascular events might have been expected. The trial was terminated for futility after median follow-up of 28 months, with 12.9% allocated evacetrapib versus 12.8% allocated placebo experiencing a primary outcome (HR: 1.01; 95% CI: 0.92 to 1.11).

#### Anacetrapib

Anacetrapib is another selective, potent, and reversible CETP inhibitor. Early studies concentrating on ensuring that there were no off-target effects on BP or aldosterone in the light of torcetrapib’s effects were reassuring. In a phase 2 study, various daily doses were assessed in the presence and absence of atorvastatin among 589 patients with primary hypercholesterolemia or mixed dyslipidemia. Results showed dose-dependent reductions in LDL-C with the highest 2 doses reducing LDL-C (Friedewald equation) by 40% and increasing HDL-C by ≤140% (30). No effect was seen on BP. The DEFINE (Determining the Efficacy and Tolerability of CETP Inhibition with Anacetrapib) study (31) adopted a Bayesian approach to allow the exclusion of a 25% increase in cardiovascular events as observed with torcetrapib. A total of 1,623 patients with stable coronary heart disease or at high cardiovascular risk and on a stable dose of statin were randomized to anacetrapib 100 mg daily or placebo. Allocation to anacetrapib increased HDL-C by 138% and apolipoprotein A1 by 47%, and decreased LDL-C (Friedewald equation) by 40% and apolipoprotein B levels by 21%. There was no effect on BP, and no safety concerns emerged over 18 months.

Unlike the 3 other CETP inhibitors, anacetrapib accumulates during treatment and has a considerably longer terminal elimination half-life. A phase 2b dose-ranging study had observed detectable anacetrapib drug levels and residual effects on lipids 8 weeks after cessation of therapy (32). Consequently, after the main treatment phase of DEFINE, blood was collected for assessment of drug and lipid levels during a 12- to 24-week off-drug reversibility phase in 1,398 patients of whom 684 had been on anacetrapib (32). After 12 weeks off anacetrapib, LDL-C (Friedewald equation) remained 19% lower among those previously on anacetrapib than on placebo, and HDL-C remained 73% higher. Plasma drug levels were about 40% of treatment phase trough levels. Among a small number studied at 2.5 and 4 years off treatment, there was still detectable drug in the plasma. It has now been demonstrated that there is accumulation of anacetrapib in adipose tissue (33). Patients who completed follow-up of DEFINE’s reversibility phase were eligible for the DEFINE 2-year extension study to assess longer-term safety and lipid effects (34). Patients resumed their previously allocated randomized treatment. Lipid effects by the end of the extension study were similar to those during DEFINE, and no safety concerns emerged.

From 2011 to 2013, 30,449 patients with stable atherosclerotic vascular disease were randomized to anacetrapib 100 mg daily or matching placebo in the REVEAL study 35, 36. Patients were provided with background atorvastatin 20 to 80 mg daily depending on previous LDL-C levels and geographical region. Baseline lipids (on atorvastatin) showed LDL-C 61 mg/dl (1.6 mmol/l), HDL-C 40 mg/dl (1.0 mmol/l), and TG of 124 mg/dl (1.4 mmol/l), and patients were followed for a median of 4.1 years. At the trial midpoint, LDL-C (measured by direct assay) was reduced by 41% (26 mg/dl [0.68 mmol/l]) but, when measured by beta quantification in a subgroup, only reduced by 17% (11 mg/dl [0.28 mmol/l]). The primary endpoint of nonfatal myocardial infarction, coronary death, or coronary revascularization was significantly reduced by 9% (95% CI: 3% to 15%) with 10.8% of those allocated anacetrapib versus 11.8% of those on placebo experiencing a first event. Little effect was seen during the first 2 years, with clear benefit emerging from then onward. A small increase in systolic BP of 0.7 mm Hg was seen on anacetrapib, and more patients developed an eGFR <60 ml/min/1.73 m^2^ than on placebo. Anacetrapib was not associated with any excess of noncardiovascular serious adverse events.

## Why Have There Been Discrepant Phase 3 Trial Results for the Different CETP Inhibitors?

Of the 4 CETP inhibitors tested in clinical outcome trials, only anacetrapib reduced cardiovascular events—why did the other trials fail to show benefit as compared to placebo? One important factor is that the effect of CETP inhibition on LDL-C, as estimated by the Friedewald equation, appears to be substantially overestimated. This issue is discussed in the next section. With regard to torcetrapib, the 5 mm Hg increase in systolic BP is likely to have outweighed any potentially beneficial effects of the lipid changes. The other 3 CETP inhibitors do also appear to increase BP, albeit to a much smaller extent.

Dalcetrapib is the weakest of the 4 CETP inhibitors and may be considered the purest test of whether raising esterified cholesterol in more mature HDL particles is beneficial. Observational studies indicate that ∼15 mg/dl (0.4 mmol/l) higher HDL-C is associated with 22% lower CHD risk (37). If HDL-C is causally (though inversely) related to CHD, then we may hypothesize that about one-half of this effect might be reversible in a short-term trial (proportionally similar to the reversibility observed with LDL-C reduction by statins over about 5 years 37, 38); that is, the expected impact of this increase in HDL-C, as was observed in Dal-OUTCOMES, might reduce CHD by about 11%. Dal-OUTCOMES was powered to detect a 15% reduction in its primary outcome. The 95% CI of the primary outcome point estimate excluded a benefit of 11%, although the trial was stopped early for futility. Two other factors may have affected this study’s power. The first is the likely delay before the effect on lipids could reasonably translate into a clinically meaningful effect on risk—with statin therapy, only about one-half of the effect is seen during the first year of treatment; second, Dal-OUTCOMES included patients within three months of an acute coronary syndrome. Such patients may be at high risk of recurrent events that are less likely to be amenable to lipid modification in comparison with patients in a more stable phase of disease. Consequently, results of Dal-OUTCOMES were inconclusive.

With regard to evacetrapib, based on observational analyses (37), the increase in HDL-C of ∼60 mg/dl (1.6 mmol/l) in ACCELERATE could be associated with ∼60% lower risk of coronary disease, of which a reasonable proportion might be reversible and, in combination with the decrease in LDL-C of ∼30 mg/dl (0.8 mmol/l), might have been expected to have reduced risk substantially more. However, if HDL-C raising has no effect on cardiovascular risk (as now suggested by multiple genetic studies) and if any benefit was therefore due to reduction in atherogenic apolipoprotein B–containing lipids alone, then the observed effect on cardiovascular risk in ACCELERATE is not inconsistent with other strands of evidence. For example, if the impact of evacetrapib on LDL-C is overestimated, in keeping with the 18% to 19% reduction in apolipoprotein B (i.e., ∼15 mg/dl [0.4 mmol/l]) observed in both REVEAL and ACCELERATE (Online Table 1), then this might only be associated with ∼9% reduction in cardiovascular risk given the likely lag phase of any benefit, a result comfortably within the CIs of ACCELERATE’s primary outcome.

In contrast to ACCELERATE, REVEAL continued for 4 years, accumulating 40% more patient years than the other phase 3 trials combined, and demonstrated a significant 9% reduction in risk in association with modest absolute reductions in non–HDL-C (17 mg/dl [0.44 mmol/l]), LDL-C (11 mg/dl [0.28 mmol/l] measured by beta quantification) and apolipoprotein B. Cardiovascular benefit only emerged in years 3 and 4. Whether ACCELERATE might have demonstrated modest cardiovascular benefit had it continued for its full duration is unclear. Evidence from genetic studies and statin trials confirms that the clinical benefit of lipid-modifying therapies is largely determined by the cardiovascular risk of the patient, duration of treatment, and the absolute reduction achieved in LDL-C (or non–HDL-C). Anacetrapib’s effect can be fully accounted for by the reduction in apolipoprotein B or non–HDL-C (Figure 2), supporting the hypothesis that there was little additional effect from raising HDL-C.

## Measurement of LDL Cholesterol During CETP Inhibitor Treatment

The relative reduction in cardiovascular disease derived from pharmacological reduction of LDL-C is well-established. For every 40 mg/dl or 1 mmol/l reduction in LDL-C, the risks of myocardial infarction, ischemic stroke, and coronary revascularization are reduced by around one-quarter after the first year. This highlights the need for accurate estimation of a treatment’s likely effect on LDL-C. Various approaches are available to estimate circulating LDL-C, namely by estimation with the Friedewald formula, measurement by means of direct detergent-based or antibody-based assays, or by beta quantification, during which TG-rich VLDL is removed, allowing accurate measurement of both LDL and HDL-C. Friedewald estimation is based on the assumption that circulating TG in the fasting state is contained in VLDL.

Recent analyses have demonstrated that, during potent CETP inhibitor therapy, both the Friedewald equation and direct assays appear to underestimate LDL-C compared with beta quantification. This underestimation appears to be predominantly seen in low to moderately low LDL-C levels (∼60 to 80 mg/dl [1.6 to 2.1 mmol/l]). Two examples from major trials of anacetrapib showed that direct LDL-C assays or calculation by the Friedewald equation provided estimates of LDL-C reduction that were approximately double the reduction by beta quantification 36, 39. By contrast, both non–HDL-C and apolipoprotein B100 appear to provide more informative measures. Data in Table 2 demonstrate the modest reductions in apolipoprotein B100 from CETP inhibition compared with the substantially larger estimated reductions in calculated or directly measured LDL-C, a discrepancy also detected in a recent genetic study (13). This underestimation appears less important at elevated LDL-C levels—in the REALIZE trial conducted in patients with familial hypercholesteremia, similar results for change in LDL-C were obtained by Friedewald equation, direct assay, and beta quantification (40).

**Table 2:** Characteristics of Phase 3 Cardiovascular Outcome Trials of CETP Inhibitors

Drug	Phase 3 Outcome Trial (Ref. #)	Type of Patient	Follow-Up (months)	Number Randomized	Baseline LDL-C (mg/dl)	Baseline HDL-C (mg/dl)	Change in LDL-C (%)∗	Change in apo-B100 (%)	Change in HDL-C (%)	Change in SBP (mm Hg)	Change in hsCRP (%)	Primary Outcome Events on Active Therapy (%)	Primary Outcome Events on Placebo Therapy (%)	HR (95% CI)
Torcetrapib†	ILLUMINATE (20)	Stable CVD	18	15,067	80	49	−28	−15	+70	+5.4	+3	6.2	5.0	1.25 (1.09–1.44)
Dalcetrapib‡	Dal-OUTCOMES (25)	ACS	31	15,871	76	42	0	0	+27	+0.6	+18	8.3	8.0	1.04 (0.93–1.16)
Evacetrapib§	ACCELERATE (29)	ACS, or stable CVD	26	12,092	81	45	−37	−19	+132	+1.2	+9	12.9	12.8	1.01 (0.91–1.11)
Anacetrapib‖	REVEAL (36)	Stable CVD	49	30,449	61	40	−41	−18	+104	+0.7	—	10.8	11.8	0.91 (0.85–0.97)

apo-B100 = apolipoprotein B100; ACS = acute coronary syndrome; CI = confidence interval; CVD = cardiovascular disease; HR = hazard ratio; SBP = systolic blood pressure; other abbreviations as in Table 1.

∗ Based on direct assay in REVEAL (17% reduction by beta quantification), Friedewald equation in ILLUMINATE and Dal-OUTCOMES, beta quantification in ACCELERATE.

† Lipid analyses at 1 year (with the exception of apo B100 [3 months]).

‡ Lipid analyses at 3 months.

§ Lipid analyses at 3 months.

‖ Lipid analyses at trial midpoint.

## Future Perspectives

Compared with routinely used nonstatin LDL-C lowering oral agents (such as ezetimibe), anaceptrapib is comparable in terms of lipid-modifying efficacy and safety (Table 3). However, it was recently announced that Merck will not pursue regulatory filing. Given that the proportional reduction of apolipoprotein B concentrations by anacetrapib is independent of baseline levels, it is possible patients with elevated atherogenic lipids may derive benefit from combination therapy including statin and anacetrapib (or anacetrapib monotherapy in patients not on a statin), but further large-scale studies of anacetrapib appear unlikely. The only ongoing outcome trial of a CETP inhibitor is the dal-GenE trial (discussed in the previous text), and it is not yet clear whether another CETP inhibitor at an earlier stage of development, TA-8995, will be entered into an outcome trial.

**Table 3:** Comparison of Anacetrapib With Nonstatin LDL Cholesterol-Lowering Agents

Class of Drugs	Medication (Ref. #)	Method and Frequency of Administration	Timing of Measurement	Change in LDL-C (%)	Change in apo B (%)	Change in HDL-C (%)	Change in Non–HDL-C (%)
Bile acid sequestrant	Cholestyramine (41)	Oral, daily	Average	−13	NA	+3	NA
PPAR alpha agonist	Fenofibrate (42)	Oral, daily	1 yr	−12	−14†	+5	NA
NPC1L1 protein inhibitor	Ezetimibe (43)	Oral, daily	1 yr	−23	−13	+1	−20
PCSK9 inhibitor	Evolocumab (44)	Subcutaneous injection, every 2–4 weeks	48 weeks	−59	−49	+8	−52
	Alirocumab (45)	Subcutaneous injection, every 2 weeks	24 weeks	−62	−54	+5	−52
ATP citrate lyase inhibitor	Bempedoic acid (46)	Oral, daily	12 weeks	−20	−12	−2	−10
CETP inhibitor	Anacetrapib (36)	Oral, daily	Trial midpoint	−17∗	−18	+104	−18

NPC1L1 = Niemann-Pick C1-Like 1; PCSK9 = proprotein convertase subtilisin/kexin type 9; other abbreviations as in Tables 1 and 2.

∗ Based on beta quantification.

† at 4 months.

## Conclusions

Evidence from both genetic studies and from the largest clinical trial of a potent CETP inhibitor, anacetrapib, confirm that inhibition of CETP yields increases in HDL-C and reductions in LDL-C, apolipoprotein B, and non-HDL-C, and that these changes yield modest cardiovascular benefit.

## References

[1] Inazu A., Brown M.L., Hesler C.B. (1990). Increased high-density lipoprotein levels caused by a common cholesteryl-ester transfer protein gene mutation. N Engl J Med.

[2] Charles M.A., Kane J.P. (2012). New molecular insights into CETP structure and function: a review. J Lipid Res.

[3] Qiu X., Mistry A., Ammirati M.J. (2007). Crystal structure of cholesteryl ester transfer protein reveals a long tunnel and four bound lipid molecules. Nat Struct Mol Biol.

[4] Lauer M.E., Graff-Meyer A., Rufer A.C. (2016). Cholesteryl ester transfer between lipoproteins does not require a ternary tunnel complex with CETP. J Struct Biol.

[5] Teslovich T.M., Musunuru K., Smith A.V. (2010). Biological, clinical and population relevance of 95 loci for blood lipids. Nature.

[6] Willer C.J., Sanna S., Jackson A.U. (2008). Newly identified loci that influence lipid concentrations and risk of coronary artery disease. Nat Genet.

[7] Webb T.R., Erdmann J., Stirrups K.E. (2017). Systematic evaluation of pleiotropy identifies 6 further loci associated with coronary artery disease. J Am Coll Cardiol.

[8] Agerholm-Larsen B., Tybjaerg-Hansen A., Schnohr P., Steffensen R., Nordestgaard B.G. (2000). Common cholesteryl ester transfer protein mutations, decreased HDL cholesterol, and possible decreased risk of ischemic heart disease: The Copenhagen City Heart Study. Circulation.

[9] Ridker P.M., Pare G., Parker A.N., Zee R.Y., Miletich J.P., Chasman D.I. (2009). Polymorphism in the CETP gene region, HDL cholesterol, and risk of future myocardial infarction: genomewide analysis among 18 245 initially healthy women from the Women's Genome Health Study. Circ Cardiovasc Genet.

[10] Johannsen T.H., Frikke-Schmidt R., Schou J., Nordestgaard B.G., Tybjaerg-Hansen A. (2012). Genetic inhibition of CETP, ischemic vascular disease and mortality, and possible adverse effects. J Am Coll Cardiol.

[11] Thompson A., Di Angelantonio E., Sarwar N. (2008). Association of cholesteryl ester transfer protein genotypes with CETP mass and activity, lipid levels, and coronary risk. JAMA.

[12] Nomura A., Won H.H., Khera A.V. (2017). Protein-truncating variants at the cholesteryl ester transfer protein gene and risk for coronary heart disease. Circ Res.

[13] Ference B.A., Kastelein J.J.P., Ginsberg H.N. (2017). Association of genetic variants related to cetp inhibitors and statins with lipoprotein levels and cardiovascular risk. JAMA.

[14] Millwood I.Y., Bennett D.A., Holmes M.V. (2018). Association of CETP gene variants with risk for vascular and nonvascular diseases among Chinese adults. JAMA Cardiol.

[15] Rader D.J., deGoma E.M. (2014). Future of cholesteryl ester transfer protein inhibitors. Annu Rev Med.

[16] Bisgaier C.L., Essenburg A.D., Minton L.L., Homan R., Blankley C.J., White A. (1994). Cholesteryl ester transfer protein inhibition by PD 140195. Lipids.

[17] Clark R.W., Sutfin T.A., Ruggeri R.B. (2004). Raising high-density lipoprotein in humans through inhibition of cholesteryl ester transfer protein: an initial multidose study of torcetrapib. Arterioscler Thromb Vasc Biol.

[18] Kastelein J.J., van Leuven S.I., Burgess L. (2007). Effect of torcetrapib on carotid atherosclerosis in familial hypercholesterolemia. N Engl J Med.

[19] Bots M.L., Visseren F.L., Evans G.W. (2007). Torcetrapib and carotid intima-media thickness in mixed dyslipidaemia (RADIANCE 2 study): a randomised, double-blind trial. Lancet.

[20] Barter P.J., Caulfield M., Eriksson M. (2007). Effects of torcetrapib in patients at high risk for coronary events. N Engl J Med.

[21] Barter P.J., Rye K.A., Tardif J.C. (2011). Effect of torcetrapib on glucose, insulin, and hemoglobin A1c in subjects in the Investigation of Lipid Level Management to Understand its Impact in Atherosclerotic Events (ILLUMINATE) trial. Circulation.

[22] de Grooth G.J., Kuivenhoven J.A., Stalenhoef A.F. (2002). Efficacy and safety of a novel cholesteryl ester transfer protein inhibitor, JTT-705, in humans: a randomized phase II dose-response study. Circulation.

[23] Luscher T.F., Taddei S., Kaski J.C. (2012). Vascular effects and safety of dalcetrapib in patients with or at risk of coronary heart disease: the dal-VESSEL randomized clinical trial. Eur Heart J.

[24] Fayad Z.A., Mani V., Woodward M. (2011). Safety and efficacy of dalcetrapib on atherosclerotic disease using novel non-invasive multimodality imaging (dal-PLAQUE): a randomised clinical trial. Lancet.

[25] Schwartz G.G., Olsson A.G., Abt M. (2012). Effects of dalcetrapib in patients with a recent acute coronary syndrome. N Engl J Med.

[26] Tardif J.C., Rheaume E., Lemieux Perreault L.P. (2015). Pharmacogenomic determinants of the cardiovascular effects of dalcetrapib. Circ Cardiovasc Genet.

[27] Nicholls S.J., Brewer H.B., Kastelein J.J. (2011). Effects of the CETP inhibitor evacetrapib administered as monotherapy or in combination with statins on HDL and LDL cholesterol: a randomized controlled trial. JAMA.

[28] Suico J.G., Wang M.D., Friedrich S. (2014). Effects of the cholesteryl ester transfer protein inhibitor evacetrapib on lipoproteins, apolipoproteins and 24-h ambulatory blood pressure in healthy adults. J Pharm Pharmacol.

[29] Lincoff A.M., Nicholls S.J., Riesmeyer J.S. (2017). Evacetrapib and cardiovascular outcomes in high-risk vascular disease. N Engl J Med.

[30] Bloomfield D., Carlson G.L., Sapre A. (2009). Efficacy and safety of the cholesteryl ester transfer protein inhibitor anacetrapib as monotherapy and coadministered with atorvastatin in dyslipidemic patients. Am Heart J.

[31] Cannon C.P., Shah S., Dansky H.M. (2010). Safety of anacetrapib in patients with or at high risk for coronary heart disease. N Engl J Med.

[32] Gotto A.M., Cannon C.P., Li X.S. (2014). Evaluation of lipids, drug concentration, and safety parameters following cessation of treatment with the cholesteryl ester transfer protein inhibitor anacetrapib in patients with or at high risk for coronary heart disease. Am J Cardiol.

[33] Krishna R., Gheyas F., Liu Y. (2017). Chronic administration of anacetrapib is associated with accumulation in adipose and slow elimination. Clin Pharmacol Ther.

[34] Gotto A.M., Kher U., Chatterjee M.S. (2014). Lipids, safety parameters, and drug concentrations after an additional 2 years of treatment with anacetrapib in the DEFINE study. J Cardiovasc Pharmacol Ther.

[35] Bowman L., Chen F., Sammons E., for the REVEAL Collaborative Group (2017). Randomized Evaluation of the Effects of Anacetrapib through Lipid-modification (REVEAL)-a large-scale, randomized, placebo-controlled trial of the clinical effects of anacetrapib among people with established vascular disease: trial design, recruitment, and baseline characteristics. Am Heart J.

[36] HPS TIMI REVEAL Collaborative Group (2017). Effects of anacetrapib in patients with atherosclerotic vascular disease. N Engl J Med.

[37] Di Angelantonio E., Sarwar N., Perry P., for the Emerging Risk Factors Collaboration (2009). Major lipids, apolipoproteins, and risk of vascular disease. JAMA.

[38] Baigent C., Blackwell L., Emberson J., for the Cholesterol Treatment Trialists Collaboration (2010). Efficacy and safety of more intensive lowering of LDL cholesterol: a meta-analysis of data from 170,000 participants in 26 randomised trials. Lancet.

[39] Davidson M., Liu S.X., Barter P. (2013). Measurement of LDL-C after treatment with the CETP inhibitor anacetrapib. J Lipid Res.

[40] Kastelein J.J., Besseling J., Shah S. (2015). Anacetrapib as lipid-modifying therapy in patients with heterozygous familial hypercholesterolaemia (REALIZE): a randomised, double-blind, placebo-controlled, phase 3 study. Lancet.

[41] (1984). The Lipid Research Clinics Coronary Primary Prevention Trial results. I. Reduction in incidence of coronary heart disease. JAMA.

[42] Keech A., Simes R.J., Barter P. (2005). Effects of long-term fenofibrate therapy on cardiovascular events in 9795 people with type 2 diabetes mellitus (the FIELD study): randomised controlled trial. Lancet.

[43] Cannon C.P., Blazing M.A., Giugliano R.P. (2015). Ezetimibe added to statin therapy after acute coronary syndromes. N Engl J Med.

[44] Sabatine M.S., Giugliano R.P., Keech A.C. (2017). Evolocumab and clinical outcomes in patients with cardiovascular disease. N Engl J Med.

[45] Robinson J.G., Farnier M., Krempf M. (2015). Efficacy and safety of alirocumab in reducing lipids and cardiovascular events. N Engl J Med.

[46] Ballantyne C.M., McKenney J.M., MacDougall D.E. (2016). Effect of ETC-1002 on serum low-density lipoprotein cholesterol in hypercholesterolemic patients receiving statin therapy. Am J Cardiol.

