# Supplementary material for: Cholesteryl Ester Transfer Protein Inhibition for Preventing Cardiovascular Events: JACC Review Topic of the Week
Source: J Am Coll Cardiol. 2019 Feb 5;73(4):477–87. doi: 10.1016/j.jacc.2018.10.072 (PMC6354546; doi:10.1016/j.jacc.2018.10.072)
Supplement: Online Table 1 [file mmc1.docx]

**Supplementary information: Inhibition of Cholesteryl Ester Transfer Protein for Preventing Cardiovascular Events: *JACC* Review Topic of the Week**

*Search strategy – literature search regarding CETP polymorphisms and cardiovascular outcomes*

On 19th September 2017, PubMed was searched using the term “CETP[All Fields] AND ("coronary heart disease"[All Fields] OR "Heart Diseases"[Mesh] OR "Myocardial Ischemia"[Mesh] OR "Coronary Disease"[Mesh]) AND ("genes"[MeSH Terms] OR "genes"[All Fields] OR "gene"[All Fields] OR "genetics"[Subheading] OR "genetics"[All Fields] OR "genetics"[MeSH Terms] OR "polymorphism, single nucleotide"[MeSH Terms] OR "polymorphism"[All Fields] OR ("single"[All Fields] AND "nucleotide"[All Fields]) OR "single nucleotide polymorphism"[All Fields] OR "snps"[All Fields] OR "Mendelian randomization"[All Fields])”

This yielded 305 studies, the abstracts of which were reviewed. The following articles were excluded:

• Animal study

• Sample size <10,000

• Study not set in general population (e.g. cohort all with CHD, diabetes)

• No abstract

• Non-English journal

• Review articles

Post screening, we included those articles that:

• reported CETP as part of a discovery study (e.g. GWAS)

• characterized the CHD associations, with some using a drug-target MR framework, including meta-analyses

*Supplementary Table 1*

| ACCELERATE, baseline LDL-c 81.4mg/dL (results at 3 months):   - Change in beta quant LDL-c: between-group difference = **-37%** - Change in Apo B: between-group difference = **-19%** - Change in non HDL-c: **not reported** |
| --- |
| REVEAL, baseline LDL-c 61mg/dL (results at midpoint):   - Change in direct LDL-c: between-group difference = **-41%** - Change in beta quant LDL-c: between-group = **-17%** - Change in Apo B: between-group = **-18%** - Change in non HDL-c: **-18%** |
| REALIZE, baseline LDL-c 130mg/dL (results at 1 yr):   - Change in direct LDL-c: between-group difference = **-44%** - Change in calculated LDL-c: between-group difference = **-42%** - Change in beta quant LDL-c: between-group = **-40%** - Change in Apo B: between-group = **-25%** - Change in non HDL-c: -**36%** |
